# Breaking the external pressure-dependent paradigm in all-solid-state batteries

**DOI:** 10.1093/nsr/nwag112

**Published:** 2026-02-14

**Authors:** Jiaxin Ma, Zhong-Shuai Wu

**Affiliations:** State Key Laboratory of Catalysis, Dalian Institute of Chemical Physics, Chinese Academy of Sciences, China; School of Materials Science and Engineering, Zhengzhou University, China; State Key Laboratory of Catalysis, Dalian Institute of Chemical Physics, Chinese Academy of Sciences, China; University of Chinese Academy of Sciences, China

All-solid-state lithium batteries (ASSLBs) are regarded as a critical technology for next-generation energy storage, offering intrinsic safety and higher energy-density potential [1]. However, translating advances in solid electrolytes into functional full-cell systems remains a challenge. A major obstacle arises from solid–solid interfacial issues, in which inadequate physical contact and heterogeneous lithium-ion transport result in high interfacial resistance and rapid capacity decay [2]. To address these constraints, most ASSLB designs rely on externally applied stack pressures often reaching several megapascals to preserve interfacial contact [3,4]. Such pressure-dependent operation significantly complicates cell assembly, elevates manufacturing costs and represents a critical bottleneck in scalable production and the practical deployment of ASSLBs.

**Figure 1.**
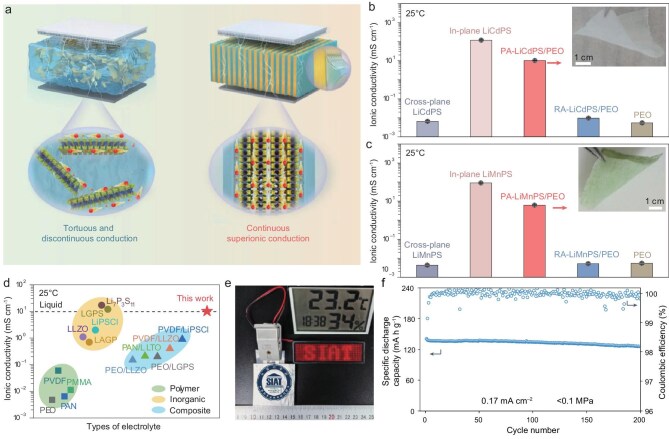
(a) Schematic illustration of the ion diffusion of a randomly dispersed electrolyte and rationally engineered lamellar composite. (b, c) Ionic conductivities of (b) Li_0.3_Cd_0.85_PS_3_ and (c) Li_0.46_Mn0._77_PS_3_ under different directions. (d) Comparison of the ionic conductivity of the as-prepared electrolyte with previously reported systems. (e) Photograph of light emitting diodes powered by a pouch cell. (f) Cycling stability of the Li||LiFePO_4_ pouch cell at pressures of <0.1 MPa. Reproduced with permission from [5].

In a recent study, Lan *et al.* proposed a paradigm shift by establishing the structural regulation of ion-transport pathways as a core design principle for solid electrolytes, moving beyond the reliance on external pressure [5]. Instead of applying external pressure to enforce interfacial contact, the authors engineered a superionic composite electrolyte, in which the mesoscale architecture ensures efficient ion transport and mechanical compliance. The rationally designed lamellar composite integrates 2D superionic sulfide nanosheets with a polymer matrix. Specifically, lithium-containing polyethylene oxide (PEO) layers are alternately stacked with perpendicularly aligned Li*_x_*M*_y_*PS_3_ (M = Cd or Mn) nanosheets, creating continuous vertical channels for rapid ion transport throughout the electrolyte thickness (Fig. 1a). The elastic PEO buffers volume fluctuations, while the flexible nanosheets and strong adhesion convert vertical stress into lateral deformation. This synergy ensures conformal solid–solid interfaces and stable ion flux without external pressure-assisted stabilization.

This design exploits the pronounced transport anisotropy of the Li_0.3_Cd_0.85_PS_3_ nanosheets (Fig. 1b and c), which exhibit ultrahigh in-plane ionic conductivities of 120 mS cm^−1^ for Li_0.3_Cd_0.85_PS_3_ and 90 mS cm^−1^ for Li_0.46_Mn0._77_PS_3_, while their through-plane conductivity remains orders of magnitude lower (∼10^−3^ mS cm^−1^). In conventional randomly dispersed composites, such anisotropy leads to discontinuous ion pathways and severe transport limitations. In contrast, the vertical alignment of these 2D nanosheets effectively redirects their intrinsically fast in-plane superionic transport along the through-plane direction, thereby establishing continuous and efficient conduction channels across the electrolyte. Consequently, the aligned composite electrolyte achieves a room-temperature ionic conductivity of 10.2 mS cm^−1^ (Fig. 1d), which is nearly three orders of magnitude higher than that of its randomly structured counterpart (∼0.0096 mS cm^−1^).

Beyond achieving high ionic conductivity, the authors also tackle the trade-off between mechanical robustness and ion transport through a bioinspired architectural strategy. Inspired by the hinge structure of clam shells, in which rigid aragonite nanowires alternate with compliant organic layers to integrate strength with flexibility, the composite electrolyte employs perpendicularly aligned layers to facilitate rapid Li^+^ transport, while the PEO layers accommodate mechanical strain through ether and hydroxyl bonding. This architecture effectively converts vertical compressive stress into lateral deformation, thereby decoupling ionic conduction from mechanical constraint and enabling continuous superionic transport without a reliance on external pressure. Owing to the homogenized lithium-ion flux and improved interfacial stability, ASSLBs incorporating this electrolyte maintain stable operation under near-zero external pressure. In Li||LiFePO_4_ pouch cells, the system delivers ∼89% capacity retention over 200 cycles at pressures of <0.1 MPa (Fig. 1e and f), demonstrating durable cycling performance without pressure-assisted stabilization.

In summary, this work establishes a clear structure–function relationship for solid electrolytes, positioning mesoscale architectural control as a factor that is equally critical to chemical composition in governing device-level behavior. In addition, this architectural principle extends far beyond lithium battery systems, offering a versatile platform for a wide array of anisotropic 2D materials, such as oxides and metal–organic frameworks, to be integrated into high-performance energy devices for multivalent-ion batteries and functional membranes. By establishing mesoscale structural control as a universal design degree of freedom, this work paves the way for next-generation high-performance, pressure-less and scalable electrochemical energy-storage devices.
